# Updates on the Role of Probiotics against Different Health Issues: Focus on *Lactobacillus*

**DOI:** 10.3390/ijms24010142

**Published:** 2022-12-21

**Authors:** Arifa Un-Nisa, Amjad Khan, Muhammad Zakria, Sami Siraj, Shakir Ullah, Muhammad Khalid Tipu, Muhammad Ikram, Myeong Ok Kim

**Affiliations:** 1 Division of Life Science and Applied Life Science (BK21 FOUR), College of Natural Sciences, Gyeongsang National University, Jinju 52828, Republic of Korea; 2 Department of Pharmacy, Faculty of Biological Sciences, Quaid-i-Azam University, Islamabad 45320, Pakistan; 3 Institute of Pharmaceutical Sciences, Khyber Medical University, Hayatabad, Peshawar 25000, Pakistan; 4 Alz-Dementia Korea Co., Jinju 52828, Republic of Korea

**Keywords:** probiotics, *Lactobacillus* probiotics, Alzheimer’s disease, depression, neurodegenerative diseases

## Abstract

This review article is built on the beneficial effects of *Lactobacillus* against different diseases, and a special focus has been made on its effects against neurological disorders, such as depression, multiple sclerosis, Alzheimer’s, and Parkinson’s disease. Probiotics are live microbes, which are found in fermented foods, beverages, and cultured milk and, when administered in an adequate dose, confer health benefits to the host. They are known as “health-friendly bacteria”, normally residing in the human gut and involved in maintaining homeostatic conditions. Imbalance in gut microbiota results in the pathophysiology of several diseases entailing the GIT tract, skin, immune system, inflammation, and gut–brain axis. Recently, the use of probiotics has gained tremendous interest, because of their profound effects on the management of these disease conditions. Recent findings suggest that probiotics enrichment in different human and mouse disease models showed promising beneficial effects and results in the amelioration of disease symptoms. Thus, this review focuses on the current probiotics-based products, different disease models, variable markers measured during trials, and evidence obtained from past studies on the use of probiotics in the prevention and treatment of different diseases, covering the skin to the central nervous system diseases.

## 1. Introduction

Probiotics are microorganisms involved in the growth and development of other microorganisms, derived from a Greek word meaning “for life” [1,2]. The well-accepted definition of probiotics was given by Fuller, according to which “Probiotics are live microbial feed supplements which beneficially affect the host animal by improving microbial balance” [3]. WHO redefines the term as “live microorganisms which when administered in adequate amounts confer a health benefit on the host” [1]. The concept of probiotics was introduced by Elie Metchnikoff in 1907. He introduced the idea that food microbes can modify the normal flora of the human body and that replacement of harmful microbes with beneficial microbes is possible. Based on this concept, the term “probiotics” was defined in different ways [4]. The term probiotic was first used by Lilly and Stillwell in 1960. In 1857, Pasteur discovered the first bacteria that were lactic acid-producing. Then in 1878, Lister also separated and recognized these lactic acid bacteria [5]. In 1889, Henry Tissier discovered Bifidobacterium and also found that these bacteria could be used to treat acute gastroenteritis caused by an imbalance of harmful microorganisms. The idea that probiotics could be friendly and used to treat certain intestinal diseases was also reported and presented by Tissier, in 1906 [6,7]. The most widely used microorganism as probiotics is *Lactobacillus*, *Bifidobacterium*, and *Saccharomyces boulardii*. *Lactobacillus* and *Bifidobacterium* are Gram-positive rods that are obligated facultative anaerobes and *S.boulardii* is a yeast [8].

Many strains of *Lactobacillus species* show their role as probiotics. The most notable of them are shown in Table 1.

*Lactobacilli* is called lactic acid-producing bacteria because it produces lactic acid as an end product of sugar fermentation by a non-respiring mode of metabolism. *Lactobacillus* belongs to *Firmicutes* phylum, class *Bacilli*, and family *lactobacillacea. Lactobacilli* are long, Gram-positive, and non-spore-forming rods that grow in a specific media called lactobacilli MRS agar media. They are nutritiously fastidious [32,33]. In adults, *Lactobacilli* localize in the oral cavity within the range of 10^3^–10^4^ CFU/g, in the ileum within the range of 10^3^–10^7^ CFU/g, and they are the dominant microorganism in the vagina while in infants’ feces their amount varies within 10^5^ to 10^8^ CFU/g [34]. The variability of the amount in humans and animals depends upon animal species, age, and locality in the gut [35]. *Lactobacillus* is a very important probiotic that is now widely used for the treatment of many disorders, as it localizes throughout the human body, including inner and outer surfaces [36].

### General Mechanism of Action of Lactobacillus Probiotics

*Lactobacilli* can perform their probiotic action by following variable mechanisms. They inhibit pathogens by microbe–microbe interaction and refurbish microbial homeostasis. This may occur as a result of antimicrobial substance production and cell-to-cell interactions. They may have an immunomodulatory role, and can either act as immunostimulators or as an immune inhibitor. Immunomodulation results by regulation of cytokine expression, modulation of dendritic cells, and Treg cells, and by effecting phagocytosis [37]. They can also maintain and modulate the functioning of the epithelial barrier by inducing mucin, preserving tight junctions, and by their anti-apoptotic effects [32]. Another mechanism by which probiotics produce a protective effect is by enhancing the level of sIgA within the intestine. The sIgA is involved in the translocation of IgA to the luminal side of epithelial cells which results in enhanced barrier functioning [38]. By attenuating NF-kβ and by enhancing IgA, probiotics up-regulate the level of anti-inflammatory cytokines and down-regulate the pro-inflammatory cytokines level, thus inducing a protective response [39]. By enhancing the levels of DCs and Treg cells, probiotics are a better, natural, and safer candidate for inflammatory diseases. By suppressing the expression of TLR, the expression of TNF-α and NF- kβ is inhibited [40]. *Lactobacilli* strain can also produce their probiotic action by exhibiting an enzymatic role, particularly β- glucosidase activity. By producing bile salt hydrolase, *Lactobacillus* species plays a vital role in bile acid metabolism [41].

**Figure 1:**
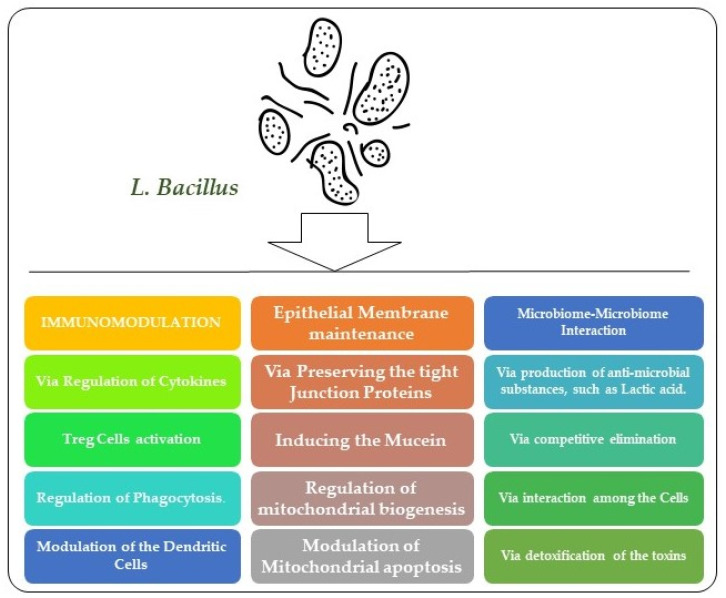
Effects of *Lactobacillus* on the maintenance of the epithelial barrier, immunomodulation, and microbe–microbe interaction. Here, we have shown that *Lactobacillus* may affect the integrity of the epithelial membrane by regulating tight junction proteins, reducing apoptotic cell death, and release of mucins. Similarly, it has immunomodulatory and regulating effects against microbe–microbe interaction.

In Figure 1, we have shown the effects of the probiotic *Lactobacillus* strain on the maintenance of the epithelial barrier, immunomodulation, and microbe–microbe interaction.

## 2. Methodological Approaches

This comprehensive review article provides insight into the role of gut microbiota (*Lactobacillus*) in the management of different diseases. An emphasis has been made on its role against neurodegenerative conditions, mainly multiple sclerosis, Alzheimer’s, and Parkinson’s diseases. The motivation for the compilation of this review article is solely based on our work on the role of probiotics against different diseases. It was a comprehensive work, where we collected reputable research articles, showing the protective potentials of *Lactobacillus* against different diseases. The articles were collected from different independent data sources (Pubmed, Web of Science, and Google Scholar), by searching the keywords “lactobacillus”, “Protective effects” “Probiotics against multiple sclerosis”, Probiotics against Alzheimer’s disease, “Probiotics against Parkinson’s disease”, and “Probiotics against metabolic disorders”

Comparisons between the *Lactobacillus*-treated and the diseased groups have been included in the manuscript. The control group was set as a standard normal group; mostly the control group was injected with physiological saline. Administration of the route of *Lactobacillus*, duration of treatment, and the model group was not considered. No duplicate and retracted articles have been included in the manuscript. The number of publications studied ranged from 200 to 250. The abstracts of all manuscripts have been collected and carefully reviewed for the compilation of this work, as outlined previously [42].

## 3. *Lactobacillus* as Probiotics in General Health

### 3.1. Effects on Gastrointestinal Tract (GIT)

*Lactobacilli* strains show good adherence to the epithelial cell layer of GIT and are thus protective. They show their effectiveness in intestinal diseases, travelers’ diarrhea, antibiotic-associated diarrhea, bowel disorders, allergy, etc. Probiotic Lactobacillus strains act in different ways by binding the epithelial cell surface of the host to minimize the harmful effects of bacterial enteric pathogens, by enhanced production of the protective mucus layer, by downregulation of inflammatory mediators, etc. [43]. They can induce the secretion and production of mucins from human epithelial cells of the intestine. This results in an increased production of mucus layer surrounding the gut that is protective and also enhances the removal of enteric pathogens [44]. Probiotic *Lactobacillus* strains also show an effective role in the prevention of adherence to the epithelial layer of the gastrointestinal tract. A study performed by Johnson-Henry KC et al. evaluated this effect and concluded that all *Lactobacillus helveticus* strains block the adherence of Escherichia coli with an epithelial barrier, which is an initiative of bacterial pathogenesis. This occurs via surface-layer proteins (slps) of the microbe [45]. Probiotic *Lactobacillus* strains can stimulate the expression of β-defensin mRNA and so have a valuable role against infectious enteritis [46,47]. The direct effect of *Lactobacillus* on the epithelial layer is also reported. Probiotic *Lactobacillus* strains decrease the production of TNF-α and inflammatory cytokines to enhance the integrity of the epithelial barrier. They may also increase the integrity and tightness of the epithelial barrier by inhibiting any kind of change in tight junction proteins of the GI tract [48]. They have a role in the reduction of apoptosis which is important to modify barrier resistance against proinflammatory cytokines [49]. Diarrhea may occur due to pathogenic microorganisms such as *Clostridium*, *Salmonella*, *Shigella*, *Rotavirus*, etc., variations in the immune system, and may also be due to physiological causes. These causes may have a link with an imbalance in human normal flora and can be treated with normal flora modifications. Approximately 50 to 80% of cases of traveler’s diarrhea are bacterial, while the remaining is due to virus and protozoa. It is reported that different *Lactobacillus* strains are effective in the reduction and prevention of traveler’s diarrhea as well as antibiotic-associated diarrhea. Randomized controlled trials performed by Hilton et al. show that ingestion of probiotic *Lactobacillus strain* is effective in reducing the daily risk of diarrhea development [50]. Different *Lactobacillus* strains, such as *L. rhamnosus*, *L. bulgaricus*, *L. acidophilus*, and *L. reuteri* along with *Bifidobacterium*, show their effectiveness in reducing the period of rotavirus infection in pediatric diarrhea [51]. Acute diarrheas in children are caused by rotavirus. A multicenter trial performed by Guandalini et al. (2000) examined the effect of oral rehydration solution containing *Lactobacillus rhamnosus* strain GG in comparison to simple ORS in pediatric diarrhea. It was concluded that administration of oral rehydration solution containing *Lactobacillus rhamnosus* strain GG in children suffering from acute diarrhea is safe. Compared to the ORS, it results in lowering the duration of the diarrhea period along with faster recovery and discharge from the hospital [52]. A meta-analysis performed by VanNiel et al. (2002) examined the safety and efficacy of probiotic *Lactobacillus* strain therapy in children suffering from acute infectious diarrhea. The results of the meta-analysis also confirm the safety, effectiveness, reduced duration, and reduction in stool per day [53]. Another probiotic strain of *Lactobacillus*, i.e., *Lactobacillus reuteri* strain, when administered with zinc showed a beneficial role in the maintenance of acute pediatric diarrhea. This was verified by a randomized, double-blind trial in 2018 [54]. Shornikova AV et al. performed a randomized trial to check the effectivity of the *Lactobacillus reuteri* strain in *Rotavirus* infection. After administration, the *Lactobacillus reuteri* strain successfully colonizes the GIT, effectively reducing the watery diarrheal duration caused by *Rotavirus* [55]. Some chronic conditions of the gastrointestinal tract involve Crohn’s disease and ulcerative colitis. The symptoms of these conditions include inflammation and diarrhea. In the case of Crohn’s disease, inflammation occurs in the colon, mucosa, and submucosa, while in the case of ulcerative colitis it remains only in the mucosa and submucosa. The combination of both of these chronic conditions is called inflammatory bowel disease (IBD). VSL # 3 sachet is a combination of 900 billion lyophilized bacteria including four strains of *Lactobacillus*, i.e., (*L. casei*, *L. plantarum*, *L. acidophilus*, and *L. delbrueckii* subsp. *bulgaricus*), three strains of *Bifidobacterium* i.e., (*B. longum*, *B. breve*, and *B. infantis*), and one strain of *Streptococcus salivarius* subsp. *thermophilus.* It was concluded that *Lactobacillus* along with other probiotics species is effective in the treatment and maintenance of the remission state of Crohn’s disease and ulcerative colitis [56]. Irritable bowel syndrome (IBS) is another chronic condition of GIT affecting the large intestine. Symptoms include abdominal pain, cramps, diarrhea, bloating, and gas. A randomized blinded trial involving 50 adults with IBS concluded that there is a beneficial and effective role of *lactobacillus Plantarum* in reducing pain in patients suffering from IBS [57].

### 3.2. Lactobacillus as Immune Modulators

*Lactobacilli* can influence the innate as well as adaptive immune system by acting as phagocytic cells, natural killer cells, and cytotoxic T cells, as a result enhancing the production of IgA antibodies and activating Toll-like receptors. This occurs by attachment with pattern recognition receptors (PRR) on immune cells as well other tissues of the intestinal epithelium. Attachment of opportunistic pathogens to epithelium is also prevented via the production of lactic acid and reactive oxygen species [58]. Different strains of probiotics show variable results based on the type of microorganism used as probiotics, dose, route of administration, and immunological condition of the patient. Different species of *Lactobacillus* are used to evaluate their effectiveness in allergic rhinitis. They show beneficial results due to their immunomodulation properties [59]. A study performed on 31 adult volunteers suffering from allergic rhinitis shows the beneficial role of *Lactobacillus paracasei*. The level of cytokines was measured in nasal fluid, and it was concluded that *Lactobacillus paracasei* can lower the amount of IL-5, IL-8, and IL-10 that are the immune markers [60]. Another report shows that oral administration of *Lactobacillus plantarum* results in the amelioration of symptoms of Birch pollen-induced allergic rhinitis by boosting Th-1 type immune response. This results in the recovery of the Th1/Th2 balance [61]. Anti-inflammatory properties of *Lactobacillus* species are also reported. By activation of Treg cells and dendritic cells, they are used to prevent and treat inflammation related diseases [62]. A study was conducted to elucidate the induction of oral tolerance in the case of rheumatoid arthritis with the help of *Lactobacillus casei*. It was concluded that this species of *Lactobacillus* proved to be effective in the potentiation of oral tolerance via up-regulation of foxp3 expression along with downregulation of Th1 type-based immune response [63]. In 2008, Jae-seon et al. elucidated the beneficial role of *L. casei* in an autoimmune disorder called rheumatoid arthritis. They deduced that *L. casei* can efficaciously lower the level of proinflammatory cytokines along with suppression of Th1 mediated cellular as well as humoral immune response [63].

### 3.3. Roles of Lactobacillus against Skin Diseases

Lactic acid bacteria can reduce the inflammatory response and hypersensitivity reactions by reducing the inflammatory mediators i.e., cytokines (Table 2).

The healing of a wound involves three main steps

- Inflammatory response
- Cell multiplication
- Remodeling of extracellular matrix

Atopic dermatitis is a chronic inflammation of the skin. It may result in a reduced antimicrobial response. *Lactobacillus* has a beneficial role in the treatment of atopic dermatitis with the help of its immunomodulatory role as shown by the following studies [64,65].

**Table 2:** Effects of L. Bacillus against different diseases.

Serial Number	Specie of *Lactobacillus*	Mechanism of Action	Result	Reference
1	*Lactobacillus Plantarum* HY7714	Reduce inflammation by regulating the expression of matrix metallopeptidases (MMP-9 and MMP-2), calprotectin, and zonulin in plasma. Enhanced IGFBP5, SERPINE1, EFEMP1, COL6A3, and SEMA3B Downregulation of MT2A, MT1E, MT1X, MT1G, and MT1F.	These results enforce that via modulating gut microbiota probiotics are beneficial for skin health.	[66]
2	*Lactobacillus fermentum* KBL375	Mast cells and eosinophils ↓ Th2 based cytokines ↓ Anti-inflammatory cytokines ↑	Useful for the treatment of atopic dermatitis via immune and metabolic modifications.	[67]
3	lipoteichoic acids obtained from *Lactobacillus Plantarum* along with *Staphylococcus aureus*	CD55 and CD59 production ↑ Downregulation of MACs Induced Th1-mediated immune response	This combination could efficaciously alleviate atopic dermatitis symptoms.	[68]
4	*Lactobacillus salivarius* LA307 along with *Lactobacillus rhamnosus* LA305	Reduced levels of serum pro-inflammatory cytokines IL-1β, IL-6, TNF-α, IL-17, IL-22 Increased levels of anti-inflammatory cytokine IL-4 and IL-10	The study suggested that probiotic is beneficial for skin elasticity and is also effective against atopic dermatitis.	[69]
5	Probiotic *Lactobacillus sakei* proBio-65	Decreased levels of pro-inflammatory cytokines such as IL-17A, IL-19, and IL-23 obtained through mRNA expression analysis	The current study revealed that this extract could be a novel treatment for treating psoriasis and an alternative to other drugs that result in several side effects.	[70]
6	*Lactobacillus acidophilus*	Transforming growth factor-β ↑ IL-12 ↑ Suppresses type-2-helper-T cell-dominant inflammation	This study shows a significant reduction in symptoms of eczema and atopic dermatitis.	[71]
7	*Lactobacillus casei*	Reduced concentration of CD8^+^ CTL Enhanced level of CD4^+^ regulatory T cells	Oral administration of L. casei may ameliorate skin inflammation by regulating the size of CD8 cell pool.	[72]

### 3.4. Metabolic Disorders

Metabolic syndrome is a cluster of conditions that occur together, increasing your risk of heart disease, stroke, and type 2 diabetes. The cardinal features of metabolic syndrome are elevated blood pressure, dyslipidemia, obesity, and an increase in fasting blood glucose. Different clinical trials show a link between human intestinal normal flora and metabolic syndromes such as obesity, diabetes, etc. A report by Larsen et al. shows that changes in gut microbiota including *Lactobacillus* species may result in improved glucose tolerance, which is the main cause of diabetes mellitus. How glucose tolerance was improved was not shown in the given article, which may be considered for future studies. It was also suggested that by regulating the gut flora there is reduced insulin resistance and overall reduced symptoms associated with diabetes mellitus type-2 [73].

## 4. Effects of Lactobacillus against Neurodegenerative Diseases

### 4.1. Microbiota–Gut–Brain (mgb) Axis

Normal flora of the GIT tract can affect and influence the functioning and development of the human brain [74]. This occurs with the help of regulatory signals among both the gut and the brain. CNS can affect the normal flora of GIT via the autonomic nervous system and hypothalamus-pituitary-adrenal association [75,76]. HPA-axis is a response system for stress that is activated by some physical or psychological stresses. As a result of stress, the hypothalamus produces and discharges a corticotrophin-releasing hormone that results in the induction of the pituitary gland and adrenal cortex to secrete adrenocorticotrophic hormone and glucocorticoids, respectively [77]. Alteration in HPA-axis, and the microbiome is one of the causes implicated and reported in affective disorders including depression, anxiety, and bipolar disorders [78]. It is reported that the altered HPA-axis can be regained by the use of Lactobacillus species. In this case, Lactobacillus reduced the permeability of the intestine and makes the HPA-axis functional [79]. Brain-derived neurotrophic factors (BDNF) are involved in brain plasticity and N-methyl-D-aspartate (NMDA) is a glutamate receptor associated with memory and synaptic plasticity. Decreased activities of both of these are reported in stressed mice. To reduce stress and anxiety-like behavior, these changes must be reversed. *Lactobacillus farciminis* treatment can result in attenuation of the hypothalamic-pituitary-adrenal (HPA) axis to reduce the stress induced in mice. As a result, it reduces the increased level of ACTH, cortisone, as well as CRF expression in the hypothalamus [80]. A study by S. Liang et al. found that *Lactobacillus helveticus* treatment shows similar results. Its chronic administration results in beneficial effects such as anti-anxiety, antidepressant, improvement in memory, and decrease in CORT and ACTH [81]. The vagus nerve also has a role in this association. *Lactobacillus* can influence the vagus nerve and results in stimulation of GABAergic receptors transcription. In this way, it induces marked changes in behavioral and psychological responses. By modulation of host immune system and by prevention of inflammatory responses *Lactobacillus* can prevent immune-mediated diseases.

### 4.2. Protective Effects of Lactobacillus against Multiple Sclerosis

An autoimmune disease triggered by aberrant T cells mediated immune response against myelin antigens. It is characterized by axonal damage, demyelination, and progressive neurological disability [82,83]. In 2018, Stephanie K. Tankou et al. evaluated the effect of probiotics including *Lactobacillus* species on gut microbiota and peripheral immune function in patients with relapsing-remitting multiple sclerosis and healthy controls. They concluded that probiotic administration results in synergistic effects with already given MS therapies by modulating immune response and also by reducing the expression of MS risk allele HLA-DQA [84]. Yuying Liu et al. in 2019 evaluated the effect of *Lactobacillus reuteri* in mice models of experimental autoimmune encephalomyelitis (EAE). This model is widely used to study multiple sclerosis and this is based on Th1 and th17 cells. They concluded that treatment with *Lactobacillus reuteri* results in a reduction of Th1/ Th17 cells and their amalgamated cytokines IFN-γ/IL-17 in EAE. They added that probiotic *L. reuteri* treatment results in changed gut microbiota to regulate immune responses in this EAE model of MS [85]. In 2018, Maya Yamashita et al. evaluated the effect of *Lactobacillus helveticus* by IP administration in a mice model of EAE and concluded that it results in reduced frequency and a clinical score of disease. Moreover, it significantly reduced IL6 production and also down-regulated Th17 differentiation and infiltration of the spinal cord, consequently relieving EAE symptoms [86]. Zohre Salehipour et al. used the combination of *Lactobacillus Plantarum* and *Bifidobacterium* species to evaluate their therapeutic potential in the EAE model of MS in 2017. It was concluded that this combination modulates immune response by enhancing anti-inflammatory cytokines while decreasing disease associated cytokines. Mononuclear infiltration of CNS, which is a pathological feature of MS, was also significantly reduced by this combinational approach. Moreover, this combination effectively diminished EAE development as well as fortifying the regulatory T- cells’ polarization [87]. Kobayashi et al. indicated in their work that *Lactobacillus Casei* administration results in up-regulation of IL-17 and IFN-γ on days 7 and 12. On day 7 levels of IL-10, CD4+ CD25+ T-reg cells up-regulated. Contradictory to this, on day 12 level of CD8+ T-cells decreased in the spleen [88]. A randomized, placebo-controlled trial was conducted by Kouchaki E et al., to evaluate the clinical and metabolic effects of probiotic capsules containing *Lactobacillus acidophilus*, *Lactobacillus casei*, *Bifidobacterium*, and *Lactobacillus fermentum* in patients with MS. They concluded that this combination showed favorable effects on the expanded disability status scale (EDSS), parameters of mental health and inflammation, insulin resistance markers, and MDA levels [89].

### 4.3. Protective Effects of Lactobacillus against Alzheimer’s Disease

**Figure 2:**
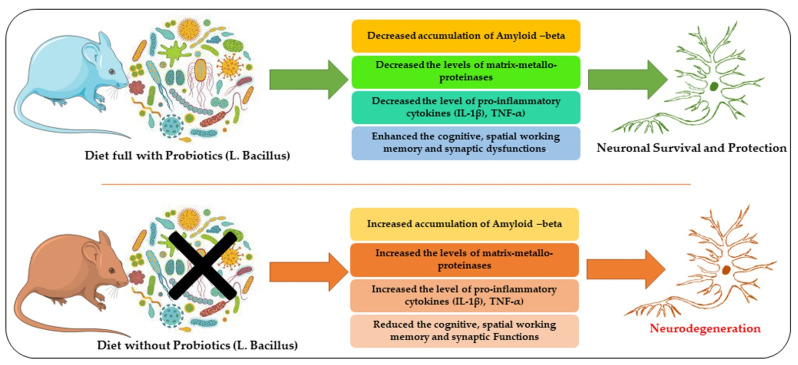
Anti-Alzheimer’s Effects of *Lactobacillus*.

AD is a neurodegenerative disorder characterized by a prominent symptom of dementia in older people with progressive loss of cholinergic neurons. The prevalence of the disease was 44 million people in 2015. Neuropathological characteristics of AD are due to the accumulation of extracellular β-amyloid protein, senile plaques, and neurofibrillary tangles intracellularly [90,91,92,93,94]. Different species of *Lactobacillus* were evaluated to find any effectiveness in AD. Most notable of these are as follows. Similarly, probiotics have shown beneficial effects on psychiatric disorders, as suggested in Figure 2 [95]. Table 3 has summarized the different species of Lactobacilli against different diseases, and its mechanisms. 

A diagram showing the rescuing effects of *Lactobacillus* against Alzheimer-associated neurodegenerative conditions. Here, we have presented that a diet with probiotics may reduce neurodegeneration by regulating the accumulation of Aβ, reducing the expression of inflammatory mediators, and enhancing cognitive functions.

### 4.4. Protective Effects of Lactobacillus against Depression

**Figure 3:**
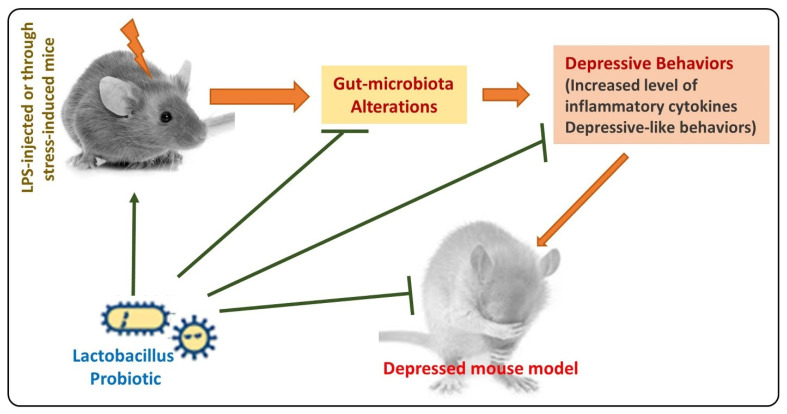
Antidepressant effects of *Lactobacillus*.

Depression is a common psychological disorder that affects 350 million people worldwide of all ages, disturbing the social functioning and quality of life of patients [108,109]. Depression is a serious, recurring, lethal, and debilitating neuropsychological disorder [110,111], characterized by loss of interest, low mood, feeling of guilt, hopelessness, change in sleep and appetite, sexual dysfunction, etc. [112]. It is the leading cause of disability and a major contributor to the global burden of disease, affecting women twice more than men (World Health Organization, 2020). Current antidepressant treatment focuses on the amending activity of a neurotransmitter in the brain. However, these treatments take weeks to produce an antidepressant effect, with severe adverse effects such as headache, agitation, nausea, sexual dysfunction, and sedation [113]. Different species of probiotics are utilized to find any effectiveness in amelioration of disease symptoms including many strains of *Lactobacillus*. Different studies of *Lactobacillus* strains are reviewed and their results are as follows: Ruining Xie et al. evaluated the effect of *Lactobacillus reuteri* in mice model of stress induced by chronic social defeat stress. They concluded that *L. reuteri* level was significantly increased in the treatment group and depressive-like behavior was also significantly treated. Moreover, *L. reuteri* resulted in a reduction of acetate and short-chain fatty acid level in the depression-induced group. The level of 5 hydroxytryptamines (5 HT) was significantly enhanced in the treatment group [114]. Abdalla M. Abdrabou et al. demonstrated the effect of *Lactobacillus acidophilus* treatment in stress-induced depression in the lab murine model. They concluded that collectively *Lactobacillus acidophilus* with citalopram (SSRI anti-depressant) significantly results in the treatment of depressive-like symptoms. This was proved based on behavior study, superoxide dismutase concentration in brain tissue, and kynurenine biomarker level by HPLC analysis [115]. Another study by Sang-Kap Han et al. showed the synergistic effect of *Lactobacillus mucosae* and *Bifidobacterium longum* in mice models of anxiety/depression induced by giving stress. They concluded that this combination could significantly alleviate symptoms of the disease by suppressing the gut microbiome dysbiosis. This conclusion was based on a behavior study on immobilization, BDNF expression, NF-κB activation in the hippocampus, stress hormone level, lipopolysaccharide, IL-6, and TNF-α levels. These are all the markers of depression [116]. Juli Choi et al. proposed that extracellular vesicles derived from *Lactobacillus Plantarum* can produce antidepressant activity in mice models of induced stress. Hence, they evaluated their hypothesis and concluded that the antidepressive effect of EV on *lactobacillus Plantarum* resulted in maintenance of the expression of neurotrophic factors, that is, BDNF, in the hippocampus [117]. Another trial with *Lactobacillus helveticus* NS8 was conducted by S.Liang et al., to check its effect on behavioral, cognitive, and biochemical alteration by chronic restrained rat model of stress. This study showed that this strain supplementation results in improvements in anxiety/depression and cognitive impairment. It was concluded based on reduced stress hormones concentration (ACTH, CORT), higher anti-inflammatory cytokines level (IL 10), increased hippocampal brain-derived neurotropic factors, and normalized serotonin and norepinephrine levels in rats [81]. Hazuki maehata et al. examined the effect of the *Lactobacillus helveticus* strain (Heat-killed) and concluded that it can significantly ameliorate the symptoms of disease in the chronic social defeat stress model [118]. Randomized, double-blinded, and placebo-controlled trials were performed using a combination of *Lactobacillus helveticus* and *Bifidobacterium longum* in seventy-nine participants with symptoms of depression. It was concluded that there was no significant difference in placebo and treatment group. Moreover, the insignificant difference was possibly due to disease severity or treatment resistance. Administration of *L. helveticus* R0052 and *B. longum* R015 in 25 human subjects for 30 days showed improvement in depression and anxiety-like behavior [119]. A double blind, placebo-controlled study was performed by Tette et al. on 423 pregnant women by using *Lactobacillus rhamnosus* HN001. Results show reduced depression scores with probiotic supplementation as compared to placebo group [120]. Another randomized, placebo-controlled, and double blind study showed that *L. Plantarum* DR7 administration for 12 weeks improved stress and anxiety-like behavior in human subjects. It also exhibited improvement in cognitive and memory functioning in stressed individuals by reducing the production of pro-inflammatory cytokines and cortisol level [121]. Overall, in most trials, depression and anxiety symptoms were ameliorated with the use of *Lactobacillus* enriched via microbe gut–brain axis and showed better results than positive control Figure 3.

The simple illustration shows the antidepressant effects of *Lactobacillus*, by regulating the gut microbiota, reducing the inflammatory cytokines, and reducing the depressive behaviors in mice.

### 4.5. Protective Effects of Lactobacillus against Parkinson’s Disease

PD is a neurodegenerative disorder that is mainly due to the loss of dopaminergic neurons in substantia nigra pars compacta, characterized by various motor as well as non-motor symptoms [122]. It is predicted that the disease will affect more than 10 million people worldwide by the year 2030 [123]. The disease is characterized by the aggregation of α-synuclein/Lewy bodies in the substantia nigra of the central nervous system. α-synuclein pathology is considered to be initiated in the enteric nervous system and then via vagus nerve spread in the central nervous system [124]. The pathology of PD is thought to be associated with oxidative stress [125], toxic agents, metabolic disorders, genetic factors, and neuroinflammation [126]. Gut microbiota plays important role in PD pathogenesis. Results of multiple studies showed that dysbiosis of gut microbes including *Lactobacillus*, *Bifidobacterium*, etc. is related to disease pathology via the gut-brain axis [127,128,129,130]. In 2020, Liao et al., evaluated the effect of *Lactobacillus Plantarum* PS128 in MPTP induced Parkinson’s disease rodent model. They concluded that supplementation with PS128 significantly alleviated the motor deficit, corticosterone elevation, nigrostriatal, and striatal dopaminergic loss. It also attenuated oxidative stress and neuroinflammation. The fecal analysis showed that the level of *L. Plantarum* was enhanced with a reduced level of *Enterobacteriaceae*. Glial cell hyperactivation was reduced. Norepinephrine and neurotrophic factors were enhanced [131].

## 5. Safety and Efficacy of *Lactobacillus*

*Lactobacillus* species used as probiotics are generally safe. Rare cases of infection and bacteremia are reported with an estimation of 0.05 to 0.4%. This should be used with caution for a patient who is severely ill or immunocompromised. Immunocompromised patients with intestinal bleeding may or may not have health benefits [132]. *Lactobacilli* isolated from a patient with endocarditis showed unusual behavior. It has higher platelet aggregation ability, collagen, and fibrinogen binding ability, and greater production of glycosidase and protease. These characteristics in turn enhance their ability to colonize vascular surfaces. The report shows that patient has recently undergone an endoscopy and it was concluded that it may result in the exposure of micro-organisms (e.g., *Lactobacillus* species) in the intestine, which could be a cause of endocarditis [133,134]. *Lactobacillus rhamnosus* also showed a risk of liver abscess. Intake of *Lactobacillus rhamnosus* by a 74-year-old woman for 4 weeks presented with a liver abscess [135]. General adverse effects offered by *Lactobacillus* strains include abdominal pain, nausea, vomiting, rash, constipation, and chest pain [136]. Seong-Tshool Hong and co-authors have presented a comprehensive review article on the recommendations on probiotics for different health issues [137].

## 6. Conclusions, Research Gap, and Future Perspectives

The aforementioned studies have suggested that gut microbiota has a profound role in the execution of several major health-related diseases, covering skin-related diseases, metabolic disorders, and neurodegenerative diseases. In all of the studies, the oral route was used for the administration of probiotics, and the duration of treatment was variable. The duration of treatment varied from 1 week to 2 years. The studies conducted on the role of probiotics in the management of several diseases have suggested that *Lactobacillus* produces strong regulating effects against different diseases. A healthy balance between host and gut microorganisms must be maintained to perform the normal physiological, metabolic, and immune functions. Rich sources of Lactobacilli include foods that contain this live bacteria, such as yogurt, or prebiotic dietary fibers found naturally in foods such as onions, garlic, and bananas, which encourage the growth of good bacteria [7]. Moreover, the *Lactobacillus* has also shown rescuing effects against AD, PD, and multiple sclerosis. In the case of AD, lactobacillus has shown significant effects against the elevated expression of Aβ, the release of inflammatory cytokines, and overall neurodegeneration. Still, reasonable work is needed to elucidate the exact effects of *Lactobacillus* against these diseases, by exploring the underlying mechanisms. The specific enzymes regulated by these probiotics may provide novel insight into the therapeutic approaches against these diseases. Collectively, the therapeutic approaches based on gut–brain health may provide relief against devastating neurodegenerative conditions such as AD and PD, and MS. Tremendous research work is needed to elucidate the mechanism of Lactobacilli in comparison with other probiotic strains.

## Figures and Tables

**Table 1:** *Lactobacillus* Species may include probiotic strains.

Species of *Lactobacillus* Used as Probiotics
*L. acidophilus* [9,10]
*L. casei* [11,12]
*L. crispatus* [13,14]
*L. gasseri* [15,16]
*L. reuteri* [17,18]
*L. rhamnosus* [19,20]
*L. plantarum* [21,22]
*L. fermentum* [23]
*L. helveticus* [24]
*L. clausii* [25,26]
*L. paracasei* [27,28]
*L. salivarius* [29]
*L. delbrueckii* [30,31]

**Table 3:** Different species of Lactobacillus showing effectiveness in different models of AD.

*Lactobacillus* Species	Model Used	Effects
* L. Fermentum *	Transgenic mice	*L. fermentum* helps in the secretion of ferulic acid which has anti-AD activity. It also helps in reducing neuroinflammation and β-amyloid plaque [96].
*L. Johnsonii* in combination with *B. Thetaiotaomicron*	Transgenic mice	This combination results in the reduction of β-amyloid plaque formation. It was concluded that this combination of probiotics along with proper exercise results in the alleviation of AD progression and beneficial effects are partly mediated by microbiome alteration [97].
*L. acidophilus*, *B. bifidum*, and *B. longum*	Male Sprague-Dawley rats	Probiotics improved learning and memory impairment. The paired-pulse facilitation ratio was also increased. This combination also proved to reduce serum levels of total cholesterol, VLDL, and triglycerides [98].
* L. acidophilus *, *L. fermentum*, *Bifidobacterium lactis*, and *B. longum*	Rats	The findings suggested that probiotics improved behavioral impairment, reduced oxidative stress by regulating the expression of malondialdehyde and superoxide dismutase, and improved cognitive dysfunctions in the AD model [99].
* Lactobacillus Plantarum* MTCC1325	Wistar rats	The ATPase system was evaluated in the hippocampus and cerebral cortex. The findings showed that lactobacillus reversed all constituents of ATPase to an almost normal level in AD-induced rats with delaying neurodegeneration [100].
* Lactobacillus acidophilus *, *Lactobacillus casei*, *Bifidobacterium bifidum*, and *Lactobacillus fermentum*	Human	The current study showed that consumption of this probiotic combination positively affects metabolic status and cognitive function in AD patients. However, it had no remarkable effect on other markers like oxidative stress, inflammation, fasting plasma glucose, and other plasma profiles [101].
Calpis sour milk whey, a *Lactobacillus helveticus*–fermented milk product	Male ddY mice	It was concluded in a current study that scopolamine-induced cognitive impairment and object recognition memory was significantly improved by oral administration of Calpis sour milk whey powder. Hence, it was suggested that it may help prevent neurodegenerative disorders, i.e., Alzheimer’s disease, and enhance learning [102].
* L. rhamnosus * as curcumin adjuvant	Mice	It was concluded that probiotics in combination with curcumin reduced the cognitive dysfunction in scopolamine-induced dementia mice. The conclusion was based on an enhanced level of antioxidant enzyme level and reduction in neuronal cell loss [103].
* L. pentosus var. Plantarum* (C29)	20 week old mice	It was concluded that treatment with C29 significantly improved memory impairment. The conclusion was based on the reversal of BDNF level suppression, DCX expression, and activation of CREB in the D-galactose-injected mice’s brains. Senescence marker p16 was also decreased along with the reduced level of inflammation markers, i.e., p-65, COX-2, p-FOXO3a, and iNOS [104].
* Lactobacillus pentosus var. Plantarum * obtained from Chinese cabbage kimchi	Mice	Probiotic supplementation inhibited cognitive dysfunction in scopolamine-injected mice by enhancing BDNF expression and p-CREB expression [105].
* L. acidophilus * with *bifidobacterium* sp.	Male Wistar rats	The findings suggested that probiotics via the gut–brain axis modulate spatial cognitive abilities and synaptic dysfunction in β-amyloid induced animal models of Alzheimer’s disease [106].
* Bifidobacterium bifidum * TMC3115 and *Lactobacillus Plantarum* 45	APP/PS1 mice	The findings suggested that supplementation of probiotics resulted in the regulation of spatial memory impairment and modified gut microbiome that further is beneficial for AD patients [107].

## Data Availability

Not applicable.
